# Thermometry on individual nanoparticles highlights the impact of bimetallic interfaces

**DOI:** 10.1038/s41467-023-38983-8

**Published:** 2023-06-27

**Authors:** Marta Quintanilla

**Affiliations:** Materials Physics Department, Universidad Autónoma de Madrid, Avda. Francisco Tomás y Valiente, 7, 28049 Madrid, Spain

**Keywords:** Materials science, Nanoscale materials

## Abstract

A new study sheds light on the impact of bimetallic interfaces in nanomaterials for heat generation using single-particle thermometry. Moving from nanoparticle ensembles to single particles is key to developing consistent knowledge of material performance and nanoscale processes, but also involves assumptions and definitions that require careful consideration.

Bimetallic nanoparticles consisting of plasmonic and catalytic components (e.g., gold and palladium) are promising materials for various applications, including photocatalysis. However, the interaction between these two components is complex and can result in new properties arising from the interface between them. Different morphological designs of bimetallic nanoparticles have been reported, with the choice of interfacial structure determining the properties of the heterometallic particle.

## Catalytic and plasmonic effects in conflict or in symbiosis?

Mixing materials with different functionalities does not necessarily yield a simple sum of their individual parts. To create nanoplatforms that excel in multiple aspects, hybrid designs incorporating two components, each optimized for a specific function, are often pursued. In doing so, we strive for symbiotic interactions. However, predicting the resulting properties can be challenging due the emergence of new phenomena arising from the interaction between the materials. This is particularly relevant in bimetallic nanoparticles containing both plasmonic and catalytic components, which are frequently proposed as enhanced photocatalytic platforms. Nanomaterials for photocatalysis are attracting significant attention because they offer the possibility of initiating reactions in remote locations, in addition to having a large active surface area per mass, a feature typical of structures in the nanometer range. This has proven effective, for example, in antibacterial surfaces^1^ or in the biomedical field, where such nanomaterials can trigger pathological effects after illumination^2^.

The microscopic characteristics of catalytic materials, such as crystal lattice spacing and defects, are intrinsically linked to their electronic and physicochemical properties. Growing these materials in close contact with a different material, induces lattice strain and differentiated lattice parameters, and inevitably impacts their catalytic performance. Plasmonic materials, on the other hand, are valued for the large extinction cross-section of their (localized) surface plasmon resonances, making them excellent candidates for light harvesting. For both catalytic and plasmonic materials, the surface actively influences their performance, underscoring the significance of the interface design in bimetallic nanomaterials. The connection between the two materials plays a crucial role in defining the properties of the hybrid particle.

**Fig. 1:**
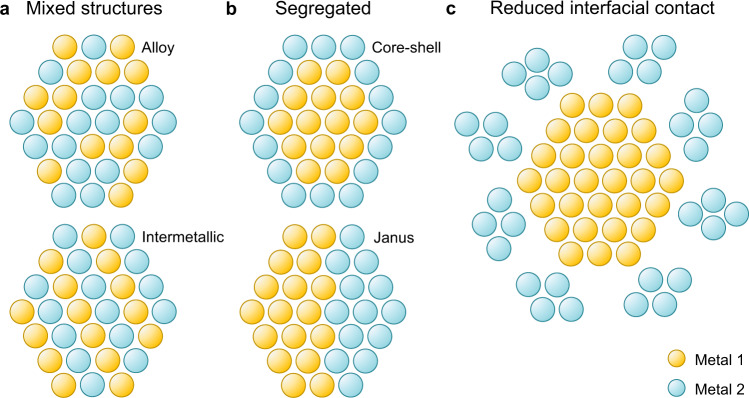
General designs of bimetallic nanoparticles. Schematic representations of the different ways in which nanoparticles composed of two different metals can be structured: (**a**) mixed structures, (**b**) segregated structures with clear interfaces, and (**c**) structures with reduced interfacial contacts. In each case, the extent of interaction between the two materials is different.

Bimetallic nanomaterials can be roughly divided into two general particle designs (Fig. 1)^3,4^: mixed structures (Fig. 1a, nanoalloys with randomly distributed components, or with an internal intermetallic order) and segregated structures with clear interfaces (Fig. 1b, core-shell or Janus designs). Recently, this scheme has been extended with alternative designs displaying a reduced interfacial contact between the structural components, e.g., core-satellites structures (Fig. 1c)^5,6^.

The choice of structure not only tunes the interfacial contact but also affects how plasmons interact with the catalytic material. Plasmonic materials’ interaction with light is complex and extends beyond light harvesting. Plasmon oscillations generate a localized (i.e., short-ranged) but strong electromagnetic field that can alter material properties through the Purcell effect. Subsequently, the energy obtained from light dissipates primarily through elastic scattering, redistributing the electromagnetic field, or heat release, elevating the local temperature.

Combining plasmonic and catalytic metals for photocatalysis holds great promise. However, the specific outcomes depend on the intricate details of the particle design due to the various physical effects involved. Since chemical reactions take place on the nanoparticle surface and reaction rates are strongly dependent on the temperature, the ability of plasmonic nanoparticles to convert a significant fraction of light into heat is of particular importance. Previous experiments on colloidal suspensions demonstrated that octopod-shaped Au/Pd nanocrystals, a morphology particularly efficient for heating, can transform between 40 and 60% of the energy obtained from light into heat^7^. Yet these are macroscopic measurements of particle ensembles in dispersion, without orientational and compositional control, which allow conclusions to be drawn about ensemble-averaged nanometric effects. However, understanding the nanoscale phenomena and surface temperatures where reactions occur necessitates the implementation of alternative techniques enabling the direct measurement of relevant parameters at the single-particle level.

## Thermometry at the nanoscale

Gargiulo et al. successfully applied a nanoscale thermometry approach based on hyperspectral measurements to address this challenge^8^. They compared two different bimetallic structures, core-shell and core-satellite, and examined their single-particle thermal characteristics. Their results highlight the potential of utilizing local heating abilities in plasmons for improved photocatalysis, complementing light harvesting and other electromagnetic interactions. Nonetheless, from an experimental point of view, single-particle thermal characterizations pose experimental considerations and requires careful analysis.

Thermal control and monitoring at the nanoscale represent a rapidly evolving field, but its progress does not follow a straightforward path. Early thermal measurements at the nanoscale, focusing on determining temperatures inside human cells and differentiating areas with distinct metabolic activity, sparked discussions regarding discrepancies between theoretical and experimental approaches^7–9^. These discussions were vital as they highlighted the need for accurate experimental techniques when alternative means to obtain to the same information are lacking. The applicability of thermodynamic concepts at the nanoscale and in reduced dimensions has also been questioned given their statistical nature^9,10^. With nanothermometry now operational and providing valuable information, the focus has shifted to standardizing protocols and defining appropriate parameters for characterizing new materials, both as thermal sensors and heaters^11^.

## Efficiency parameters for photothermal heat generation

Macroscopic measurements of photothermal agents commonly refer to heating efficiency, representing the percentage of energy converted into heat from the total energy absorbed by the nanoparticle^12,13^. This relative measurement allows comparisons between samples from different laboratories and experimental setups, as it is independent of illumination dose, heat dissipation characteristics, and heater concentration in the colloidal dispersion. If instead the temperature reached was used as a descriptive parameter of photothermal activity, a comparison would not be possible because this final temperature strongly depends on many experimental variables. However, heating efficiency is not a perfect parameter as it does not provide insight into how effectively a nanoparticle extracts energy from light. Borrowing from the concepts of photovoltaics, the heating efficiency can be considered as an internal efficiency, while an external efficiency would also take into account the available energy that does not interact with the nanoparticle. In addition, for some applications it may be useful to define an efficiency parameter that accounts for the mass of material needed to achieve a goal, for instance, a molar ratio^6^.

With the advent of investigations on single-particle level, it is time to extend the debate to this field. Gargiulo and co-workers use an alternative parameter, the photothermal coefficient, to quantify the photothermal ability of nanoparticles^8^; carefully considering all the approximations needed to arrive at a reliable result. This coefficient correlates excitation irradiance with the achieved temperature, which is both intuitive and practical, and avoids any dependence on irradiance. While it may not be optimal for macroscopic measurements due to its dependence on particle concentration, this limitation does not affect single-particle measurements. This leaves only heat dissipation as possible experimental confounding factor that could limit future comparisons with other materials. For macroscopic measurements, this would be a major disadvantage, as details such as the material of the cuvette or the exact surface area of the solution in contact with it all matter. In contrast, only the local environment plays a role in single-particle measurements, which in this case consists primarily of water. The authors demonstrate that the distance over which temperature increases is shorter than 100 nm, suggesting that heat dissipation properties beyond this range may not require consideration^8^. Then, again, the fact that measurements are single particle simplifies the situation when it comes to find a solid parameter to describe the system. Thus, as long as particles are appropriately separated, the single-particle nature of the measurements simplifies the description of the system, and the photothermal coefficient serves as a robust parameter to define single-particle photothermal properties. Notwithstanding, the photothermal coefficient wouldn´t work for nanoparticle ensembles and collective photothermal heating effects. As new experimental approaches are developed, alternative efficiency parameters may emerge, targeted to a specific application, or normalized to emphasize a specific aspect, but the description offered here for single-particle thermometry will remain valid and complete.
